# Latter-stage preclinical developmental work on PL69/DM1157

**DOI:** 10.1186/1475-2875-13-S1-P70

**Published:** 2014-09-22

**Authors:** David Peyton

**Affiliations:** 1 Portland State University, Portland, OR, USA; 2 DesignMedix, Inc., Portland, OR, USA

## 

The drug class we originally termed ‘Reversed Chloroquines’ has been assessed, via SAR, to select a candidate for preclinical evaluation. Such molecules were originally designed to function like the late-20th century Gold-standard, chloroquine, but with an appendage that intentionally inhibits resistance. Thus, PL69/DM1157 was subjected to screening, beginning with potency against many laboratory-adapted strains of chloroquine-resistant *P. falciparum* and *in vivo* efficacy in mice. PL69/DM1157, having structural features in common with chloroquine, might have cardiac effects, so we evaluated for hERG interaction, but more rigorously in a guinea pig electrocardiogram model. The results indicated the cardiac safety to be similar to chloroquine.

Academic collaborators have subjected PL69/DM1157 to clinical isolates of highly resistant *P. falciparum*, as well as to *P. vivax* strains. A chloroquine-resistant strain of *P. falciparum* was also subjected to PL69/DM1157 pressure for over two years in an unsuccessful attempt to increase IC_50_.

The project has now progressed through off-target evaluations, *in vitro* toxicity assessments, and rat preclinical toxicity tests. The molecule has been synthesized under GLP certification, without chlorinated solvents or any chromatographic steps to >99% purity, at sufficient scale to permit final toxicity evaluation in a second species, as well as in a Phase-I clinical trial, using the same batch.

This work also demonstrates how collaboration between a university and a start-up company can be an alternative pathway to bring a neglected-disease drug through the necessary drug development steps. The experience gained through this and other malaria work has enabled the company to begin collaborative work on drug-resistant bacterial diseases as well.

